# *Cardinium* symbiosis as a potential confounder of mtDNA based phylogeographic inference in *Culicoides imicola* (Diptera: Ceratopogonidae), a vector of veterinary viruses

**DOI:** 10.1186/s13071-020-04568-3

**Published:** 2021-02-08

**Authors:** Jack Pilgrim, Stefanos Siozios, Matthew Baylis, Gert Venter, Claire Garros, Gregory D. D. Hurst

**Affiliations:** 1 Faculty of Health and Life Sciences, Institute of Infection, Veterinary and Ecological Sciences, University of Liverpool, Liverpool, UK; 2 Health Protection Research Unit in Emerging and Zoonotic Infections, Liverpool, UK; 3 Agricultural Research Council- Onderstepoort Veterinary Research, Pretoria, South Africa; 4 Department of Veterinary Tropical Diseases, University of Pretoria, Pretoria, South Africa; 5 ASTRE, University of Montpellier, Cirad, INRA, Montpellier, France; 6 Cirad, UMR ASTRE, Montpellier, 34398 France

**Keywords:** Cardinium, Endosymbiont, Symbiosis, COI, Culicoides

## Abstract

**Background:**

*Culicoides imicola* (Diptera: Ceratopogonidae) is an important Afrotropical and Palearctic vector of disease, transmitting viruses of animal health and economic significance including African horse sickness and bluetongue viruses. Maternally inherited symbiotic bacteria (endosymbionts) of arthropods can alter the frequency of *COI* (cytochrome c oxidase subunit I) mitochondrial haplotypes (mitotypes) in a population, masking the true patterns of host movement and gene flow. Thus, this study aimed to assess the mtDNA structure of *C. imicola* in relation to infection with *Candidatus* Cardinum hertigii (Bacteroides), a common endosymbiont of *Culicoides* spp.

**Methods:**

Using haplotype network analysis, *COI* Sanger sequences from *Cardinium*-infected and -uninfected *C. imicola* individuals were first compared in a population from South Africa. The network was then extended to include mitotypes from a geographic range where *Cardinium* infection has previously been investigated.

**Results:**

The mitotype network of the South African population demonstrated the presence of two broad mitotype groups. All *Cardinium*-infected specimens fell into one group (Fisher’s exact test, *P* = 0.00071) demonstrating a linkage disequilibrium between endosymbiont and mitochondria. Furthermore, by extending this haplotype network to include other *C. imicola* populations from the Mediterranean basin, we revealed mitotype variation between the Eastern and Western Mediterranean basins (EMB and WMB) mirrored *Cardinium*-infection heterogeneity.

**Conclusions:**

These observations suggest that the linkage disequilibrium of *Cardinium* and mitochondria reflects endosymbiont gene flow within the Mediterranean basin but may not assist in elucidating host gene flow. Subsequently, we urge caution on the single usage of the *COI* marker to determine population structure and movement in *C. imicola* and instead suggest the complementary utilisation of additional molecular markers.

**Graphical Abstract:**

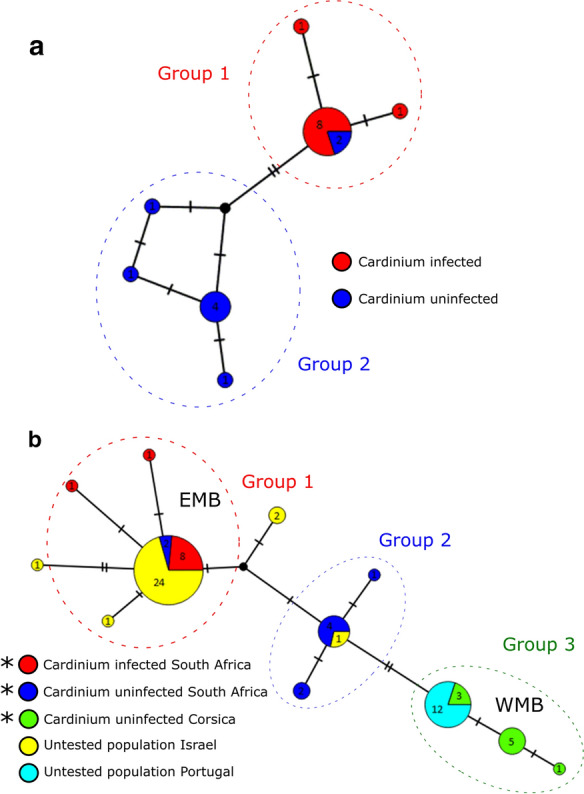

## Background

*Culicoides* spp. (Diptera: Ceratopogonidae) are a group of tiny biting midges responsible for the spread of several pathogens to animals and humans, including African horse sickness, Schmallenberg, bluetongue and Oropouche viruses. An important vector species, *Culicoides imicola,* was initially considered to be limited to Afrotropical regions but recent genetic analysis suggests this species has been present in the Mediterranean basin since the Late Pleistocene or Early Holocene [1]. The movement of *C. imicola* at the northern fringes of the Mediterranean basin is likely driven by wind events which have led to recurrent incursions of the species into mainland Europe [2]. Thus, the tracking of *C. imicola* through population genetics and climatic modelling is important in establishing where potential future outbreaks of midge-borne viruses may occur.

Mitochondrial DNA (mtDNA) markers have previously been used in phylogeographic studies to detect biodiversity and dispersal events of *C. imicola* populations [1–3]. However, mtDNA-based inferences can be confounded by the linkage disequilibrium (co-inheritance) of heritable bacteria (endosymbionts) and host mtDNA. As endosymbionts enter a naïve population, selective sweeps of the bacteria can occur and with them the homogenisation of linked mtDNA haplotypes, leading to the apparent absence of biodiversity [4]. Conversely, variation in endosymbiont presence over space can lead to a perceived genetic population structure when none exists. Subsequently, inferences from these studies can only be interpreted in the context of any history of infection with endosymbiotic bacteria.

In this study, we examine the relationship between mtDNA in *C. imicola* and the heritable endosymbiont *Candidatus* Cardinium hertigii (Bacteroidetes). *Cardinium* is present in several biting midge species, including *C. imicola*, although *Cardinium*’s significance in *Culicoides* biology is unknown [5–7]. As *Cardinium* infection could have implications for inferring movement of *C. imicola* into naïve geographic areas through mtDNA marker analysis, we analysed associations between mitochondrial haplotypes (mitotypes) and *Cardinium* infection in this important vector species.

## Methods

One hundred and six *C. imicola* individuals were collected between 2015 and 2016 using light traps from two countries: France (location: Corsica) and South Africa (location: Onderstepoort). Amplification of the host *COI* gene [8] and the *Cardinium* Gyrase B *(GyrB*) gene [7] was undertaken by conventional PCR assay. DNA extracts which passed quality control through successful *COI* amplification but were negative for *GyrB* were then screened with a more sensitive nested PCR assay (cycling conditions and primer sequence information in Additional file 1: Table S1). PCR assays consisted of a total of 15 μl per well, comprised of 7.5 μl GoTaq^®^ Hot Start Polymerase (Promega), 5.1 μl nuclease free water, 0.45 μl forward and reverse primers (concentration 10 pmol/μl) and 1.5 μl DNA template. PCR products were separated on 1% agarose gels stained with Midori Green Nucleic Acid Staining Solution (Nippon Genetics Europe). *COI* amplicons identified by gel electrophoresis were purified enzymatically (ExoSAP) before being sent for sequencing through the Sanger method (GATC Biotech AG, Konstanz, Germany). Chromatograms were then visually assessed for correct base calls. Accession numbers for *COI* sequences generated in this study are LR877434–LR877461.

We then investigated possible associations of the endosymbiont *Cardinium* with *C. imicola* mitotype distribution through haplotype network analysis. To this end, haplotype networks were constructed, with analysis and visualisation undertaken using the TCS haplotype network algorithm generated in PopART v1 [9]. The networks included *C. imicola* mitotypes from this study (South Africa and Corsica) as well as mitotypes detected from the Iberian Peninsula and Israel ([3]; Portugal *n* = 12, Israel *n* = 29; accession numbers: AF078098–AF078100, AF080531-AF080535, AJ549393–AJ549426). The Iberian population is known to be free from *Cardinium*, whereas that of Israel is known to carry *Cardinium* [5, 6]. To investigate associations between endosymbiont and mitotype, the mtDNA haplotype group and *Cardinium* infection status of *C. imicola* individuals within and between populations were compared using Fisher’s exact test (significance cut-off *P *< 0.001) in RStudio version 1.3 [10]. A Group was defined as a cluster of mitotypes with no more than one SNP difference between at least one other mitotype within the cluster.

## Results

**Fig. 1:**
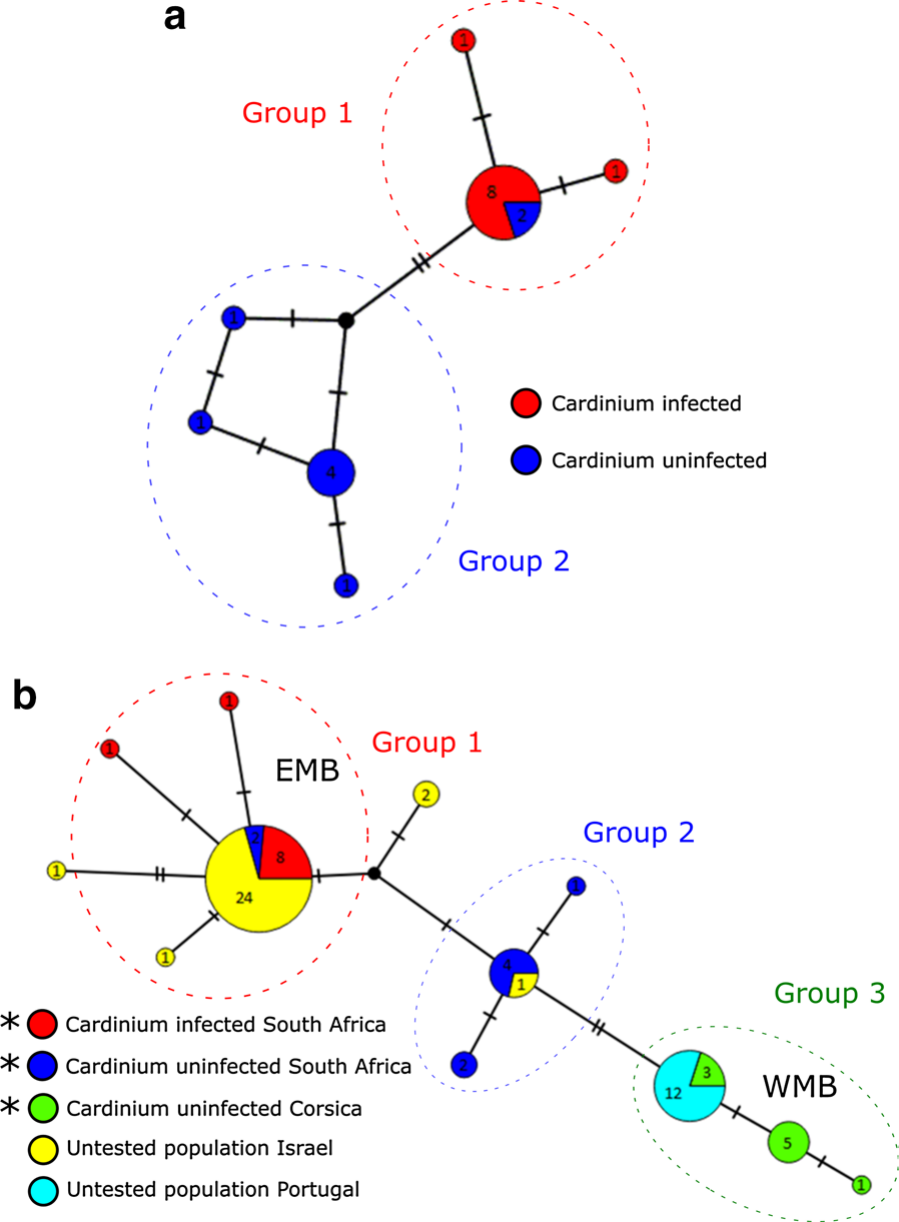
*Culicoides imicola* mtDNA haplotype networks **a** mtDNA haplotype network of *Cardinium*-infected and -uninfected *Culicoides imicola* from a single site in Onderstepoort, South Africa; based on a 517 bp *COI* sequence. **b** mtDNA haplotype network of *Culicoides imicola* from sites spanning South Africa and the Mediterranean basin; based on a 451 bp *COI* sequence. Haplotype networks were generated using the TCS network algorithm in PopART v1.7 (9). Numbers within circles represent the numbers of individuals designated to each haplotype. The numbers of substitutions separating haplotypes are indicated by dashes. A group is defined as a cluster of mitotypes with no more than one SNP difference between at least one other mitotype within the cluster. *EMB* Eastern Mediterranean Basin, *WMB* Western Mediterranean Basin. *Indicates mtDNA haplotypes generated in this study

To confirm individuals in this study were not cryptic species of *C. imicola* missed by morphological identification, a distance estimation of mtDNA barcodes was assessed giving a minimum identity of > 98%, consistent with all individuals belonging to the same species. *Cardinium* infection was observed in the South African population (10/33 individuals) but not in Corsican populations (0/73 individuals) (Table 1). The additional nested screening revealed no evidence of low-titre infections. A *C. imicola* mitotype network (Fig. 1a) of the single South African population, containing both *Cardinium*-infected and uninfected individuals, showed the presence of seven mtDNA haplotypes split into two broad mitotype groups. *Cardinium* infection status and these general mitotype groupings were associated, with all *Cardinium*-infected specimens falling in one group (Fisher’s exact test, *P *= 0.00071 [OR: Infinity; CI 3.27–Infinity]).

**Table 1:** *Cardinium* screening results using conventional and nested *GyrB* PCR assays

Species	Collection site	Year	Number of *Cardinium*-positive individuals by conventional PCR	Number of *Cardinium*-positive individuals by nested PCR
*C. imicola*	Corsica (Site 1)	2015	0/46	0/46
	Corsica (Site 2)		0/27	0/27
	South Africa (Onderstepoort)	2016	10/33	10/33

Extending the haplotype network to include *C. imicola* populations from the Western Mediterranean Basin (WMB; Corsica and Portugal) and the Eastern Mediterranean Basin (EMB; Israel) led to 12 haplotypes being observed with 0–1.77% range of divergence over a 451 bp region. Further to this, 24/29 Israeli (EMB) haplotypes clustered within the main *Cardinium*-infected mitotype observed in South Africa, whereas the Portuguese (WMB) haplotypes all clustered within an uninfected Corsican mitotype (Fig. 1b). Grouping mitotypes of known infection status within one SNP of others within a cluster defined three groups, and again all *Cardinium*-infected specimens fell in one group (Fisher’s exact test, *P* < 0.0001 [OR: NA; CI NA]).

## Discussion

Insect endosymbionts are generally considered in the context of driving diverse and extreme host phenotypes, such as reproductive manipulations and virus blocking [11, 12]. However, their role in confounding interpretations of mtDNA structure is also of importance [4]. As both endosymbiont and mitochondria are maternally inherited together, a selective sweep of *Cardinium* is likely to have led to our observed concordance of *C. imicola* mitotype and *Cardinium* (Fig. 1a). Null models of endosymbiont-mtDNA dynamics predict three phases: first, the symbiont sweep, during which the endosymbiont-associated mitotype increases in frequency and the infected and uninfected individuals have different associated mitotypes. This is followed by the mitotype of infected individuals becoming increasingly represented in uninfected individuals where the endosymbiont fails to transmit from mother to offspring. In the final phase, mutation re-establishes diversity in the mitotype pool. Although a structure is observed between infection status and mtDNA haplotype, the presence of two uninfected individuals in the main infected haplotype in *C. imicola* from South Africa is consistent with an endosymbiont showing imperfect vertical transmission, generating uninfected individuals from lineages with the dominant *Cardinium*-infected mitotype.

Although the infection status of the Portuguese and Israeli individuals included in the extended haplotype network (Fig. 1b) has not been directly assessed, the recent Pagès [6] screening for *Cardinium* found no indication of *C. imicola* infection in the Iberian Peninsula, and we observed no infections in our Corsican population. Despite this, others [5] have observed the endosymbiont to be common in Israel, indicating infection heterogeneity exists between the EMB and WMB. In light of this, mitotype variation between the EMB and WMB mirrors infection heterogeneity between *C. imicola* populations from the EMB and WMB. Thus, the mtDNA structure may be explained by the dispersal of *Cardinium*-infected individuals exclusively into the EMB.

The matrilineal subdivision between *Cardinium*-infected and -uninfected *C. imicola* (Fig. 1b) is similar to observations by previous studies [3, 13] which noted a remarkable divergence between *C. imicola* mitotypes from the EMB and WMB. This observation suggests that the linkage disequilibrium of *Cardinium* and mitochondria reflects endosymbiont gene flow within the Mediterranean basin but may not assist in elucidating host gene flow. For example, if dispersal events occur involving *Cardinium*-infected *C. imicola*, selective sweeps of the endosymbiont may erase any previous biodiversity in this marker. This is reminiscent of similar patterns observed in other insects with endosymbiont infections: the mosquito vector, *Culex pipiens* [14], the parasitoid wasp, *Nasonia vitripennis* [15], and the ladybird, *Adalia bipunctata* [16]. In all these cases, the frequency of mtDNA haplotypes are more closely associated with the endosymbionts in a population rather than geography.

## Conclusions

The haplotype networks produced in this study (Fig. 1) suggest *Cardinium* infection in *Culicoides* is another example where the presence of an endosymbiont impacts mtDNA-based inference of population history. Previous work had indicated Israeli and Southern Africa samples grouped together on analysis of mtDNA variation but were distinct on analysis of microsatellite variation [1]. We conclude that association of *Cardinium* and mtDNA provides a likely explanation for this discordance, and these data support the general importance of using multiple loci (e.g. microsatellites and nuclear markers) in phylogeographic analysis.

## Supplementary Information

**Additional file 1: Table S1.** Primer attributes and PCR conditions.

## Data Availability

Accession numbers for *COI* sequences generated in this study are LR877434–LR877461.

## Abbreviations

EMB: Eastern Mediterranean Basin
WMB: Western Mediterranean Basin
COI: Cytcochrome c oxidase subunit I
