# Correction to: Cardinium symbiosis as a potential confounder of mtDNA based phylogeographic inference in Culicoides imicola (Diptera: Ceratopogonidae), a vector of veterinary viruses

**DOI:** 10.1186/s13071-021-04653-1

**Published:** 2021-03-01

**Authors:** Jack Pilgrim, Stefanos Siozios, Matthew Baylis, Gert Venter, Claire Garros, Gregory D. D. Hurst

**Affiliations:** 1 Faculty of Health and Life Sciences, Institute of Infection, Veterinary and Ecological Sciences, University of Liverpool, Liverpool, UK; 2 Health Protection Research Unit in Emerging and Zoonotic Infections, Liverpool, UK; 3 Agricultural Research Council- Onderstepoort Veterinary Research, Pretoria, South Africa; 4 Department of Veterinary Tropical Diseases, University of Pretoria, Pretoria, South Africa; 5 ASTRE, University of Montpellier, Cirad, INRA, Montpellier, France; 6 Cirad, UMR ASTRE, 34398 Montpellier, France

## Correction to: Parasites Vectors 14:100 (2021) https://doi.org/10.1186/s13071-020-04568-3

Following publication of the original article [1], it was brought to our attention the Conclusions of the Abstract had been erroneously omitted from the PDF version of the article.

The original article has since been corrected.

The publisher apologizes for any inconvenience caused by this technical error.
