# Supplementary material for: Cardinium symbiosis as a potential confounder of mtDNA based phylogeographic inference in Culicoides imicola (Diptera: Ceratopogonidae), a vector of veterinary viruses
Source: Parasit Vectors. 2021 Feb 8;14:100. doi: 10.1186/s13071-020-04568-3 (PMC7869521; doi:10.1186/s13071-020-04568-3)
Supplement: Supplementary file 1 — Additional file 1: Table S1. Primer attributes and PCR conditions. [file 13071_2020_4568_MOESM1_ESM.docx]

**Table S1: Primer attributes and PCR conditions**

**Table S1.1.** *COI* and *Gyrase B* gene primer attributes.

| **Target** | **Primer name** | **Sequence (5’-3’)** | **Tm (°C)** | **Size (bp)** | **Reference** |
| --- | --- | --- | --- | --- | --- |
| Cytochrome oxidase subunit 1 (*COI*) | LCO2198 | GGTCAACAAATCATAAAGATATTGG | 51 | 708 | [1] |
|  | HCO1490 | TAAACTTCAGGGTGACCAAAAAATCA | 56 |  |  |
|  | C1‐J‐1718 | GGAGGATTTGGAAATTGATTAGT | 52 | 523 | [2] |
|  | C1‐N‐2191 | CAGGTAAAATTAAAATATAAACTTCTGG | 50 |  |  |
| Gyrase B | gyrB23F | GGAGGATTACATGGYGTGGG | 60 | 1368 | [3] |
|  | gyrB1435R | GTAACGCTGTACATACACGGCATC | 60 |  |  |
|  | gyrBnest212F | AAGGCAACCCTATGCACCAA | 59 | 347 | This study |
|  | gyrBnest654R | GGYCTTAGTTTGCCCTTCAAATTG | 59 |  |  |

**Table S1.2.** PCR cycling conditions

| **Target** | **Initialisation** | **Denaturation, annealing, extension** | **Final extension, hold** | **Number of cycles** |
| --- | --- | --- | --- | --- |
| Cytochrome oxidase subunit 1 (COI) | 95°C/5min | 95°C/30s, 50°C/1min, 72°C/1min | 72°C/7min, 15°C/∞ | 35 |
| Gyrase B (Conventional) | 95°C/5min | 95°C/30s, 55°C/1min, 72°C/1min30s | 72°C/7min, 15°C/∞ | 35 |
| Gyrase B (Nested) | 95°C/5min | 95°C/25s, 55°C/30s, 72°C/45s | 72°C/7min, 15°C/∞ | 35 |

**References**

1. Folmer O, Black M, Hoeh W, Lutz R, Vrijenhoek R. DNA primers for amplification of mitochondrial cytochrome c oxidase subunit I from diverse metazoan invertebrates. Mol Mar Biol Biotechnol. 1994;3:294–9.

2. Dallas JF, Cruickshank RH, Linton YM, Nolan D V., Patakakis M, Braverman Y, et al. Phylogenetic status and matrilineal structure of the biting midge, *Culicoides imicola*, in Portugal, Rhodes and Israel. Med Vet Entomol. 2003;17:379–87.

3. Lewis SE, Rice A, Hurst GDD, Baylis M. First detection of endosymbiotic bacteria in biting midges *Culicoides pulicaris* and *Culicoides punctatus*, important Palaearctic vectors of bluetongue virus. Med Vet Entomol. 2014;28:453–6.
